# Pathological identification of HER2-low breast cancer: Tips, tricks, and troubleshooting for the optimal test

**DOI:** 10.3389/fmolb.2023.1176309

**Published:** 2023-04-03

**Authors:** Elham Sajjadi, Elena Guerini-Rocco, Elisa De Camilli, Oriana Pala, Giovanni Mazzarol, Konstantinos Venetis, Mariia Ivanova, Nicola Fusco

**Affiliations:** ^1^ Department of Oncology and Hemato-Oncology, University of Milan, Milan, Italy; ^2^ Division of Pathology, IEO, European Institute of Oncology IRCCS, Milan, Italy

**Keywords:** breast cancer, HER2, HER2-low, pathology, precision medicine, immunohistochemistry, *in situ* hybridization

## Abstract

The introduction of novel anti-HER2 antibody-drug conjugates (ADC) for the treatment of HER2-low breast cancers has transformed the traditional dichotomy of HER2 status to an expanded spectrum. However, the identification of HER2-low (i.e., immunohistochemistry (IHC) score 1 + or IHC score 2+, without gene amplification) tumors is challenged by methodological and analytical variables that might influence the sensitivity and reproducibility of HER2 testing. To open all possible therapeutic opportunities for HER2-low breast cancer patients the implementation of more accurate and reproducible testing strategies is mandatory. Here, we provide an overview of the existing barriers that may trouble HER2-low identification in breast cancer and discuss practical solutions that could enhance HER-low assessment.

## Introduction

**FIGURE 1:**
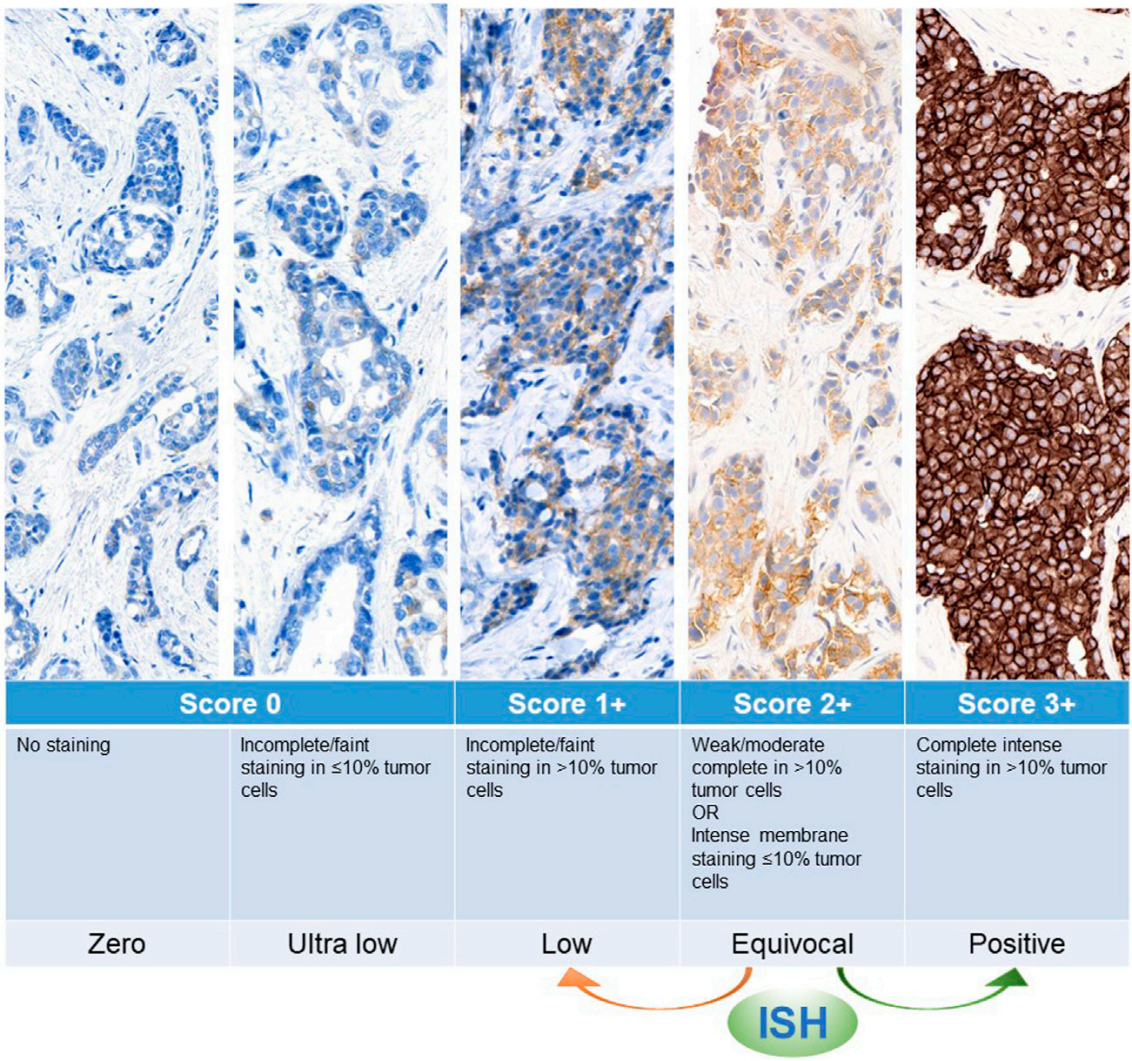
Graphical representation of HER2 expression spectrum, with score definition and interpretation according to ASCO/CAP guidelines. ISH, *in situ* hybridization.

HER2 testing is a standard procedure for all new breast cancer diagnoses, as well as in case of tumor progression and/or residual tumor after neoadjuvant treatment (Venetis et al., 2022; Pondé et al., 2018; Viale and Fusco, 2022; Fusco et al., 2022). This analysis relies on a combination of immunohistochemistry (IHC) and *in situ* hybridization (ISH). In particular, IHC detects the expression and intensity of HER2 protein on the cell membrane by a three-tier scoring system (from score 0 to score 3+) Table 1; Figure 1, while ISH detects the presence of gene amplification using HER2 and CEP17 probes (Sajjadi et al., 2022). To date, HER2+ breast cancer patients are defined as IHC score 3 + or score 2+ with a positive ISH, and subsequently qualified for anti-HER2 targeted therapy (Pondé et al., 2018; Wolff et al., 2018). Based on the DESTINY-Breast04 trial, metastatic breast cancer (mBC) with low levels of HER2 expression (i.e., IHC score 1 + or score 2+/ISH-negative) could benefit of the new anti-HER2 antibody-drug conjugate (ADC) Trastuzumab Deruxtecan (Modi et al., 2022). This drug contains a strong chemotherapeutic payload that is guided against tumor cells even with the presence of low traces of HER2 positivity thanks to the so-called “bystander effect” (Zhang et al., 2020; Venetis et al., 2022). The precise identification of HER2-low, and in particular the discrimination of score 1 + vs. score 0, is impacted by methodological and analytical variables (Sajjadi et al., 2022). In this respect, several studies are ongoing for the development of new technologies and implementation of dedicated guidelines that could provide more comprehensive and accurate assessments of HER2 status in view of HER2-low mBC. Among novel methods, artificial intelligence (AI)-based digital pathology has the potential to complement the traditional pathological analysis and to improve the accuracy of HER2 testing. In this mini review we will discuss all these challenges and opportunities, providing practical suggestions for HER2 testing, and “old” but at the same time “new” task in pathology.

**TABLE 1:** HER2 scoring by immunohistochemistry according to the expanded spectrum of positivity (ASCO/CAP 2021).

Membrane staining pattern	Tumor cells	Score	Classical category	Expanded spectrum
Intense, complete	>10%	3+	HER2+	HER2+
Weak-to-moderate, complete	>10%	2+	HER2+ (if ISH+)	HER2+ (if ISH+)
			HER2- (if ISH-)	HER2-low (if ISH-)
Faint/barely perceptible, incomplete	>10%	1+	HER2-	HER2-low
Faint/barely perceptible, incomplete	≤10%	0	HER2-	HER2 Ultra low
No staining			HER2-	HER2-zero

Abbreviations: IHC, immunohistochemistry; ISH, *in situ* hybridization; HER2, human epidermal growth factor receptor 2.

## Current challenges: how far from the perfect test?

The identification of low HER2 expression levels is not a trivial task because it relies on multiple methodological and analytical variables (Table 2). These variables might trouble the testing sensitivity and reproducibility, particularly for the discrimination between HER2-low score 1+ and “HER2-zero” (i.e., IHC score 0), which comprises also the subset of “HER2 ultra-low” (i.e., score 0 with incomplete and faint staining in ≤10% of tumor cells) (Sajjadi et al., 2022). The tissue sample handling and processing remains a crucial task, mainly subjected to the “garbage-in-garbage-out” paradigm (Perez et al., 2014). Variables such as fixation, antigen retrieval, antibody clones, reaction time, temperature, and substrate concentration can all influence the IHC staining intensity (Sapino et al., 2013; Zhang et al., 2021). Additionally, the choice of staining methodology, particularly antigen retrieval, and the availability of different antibody clones with varying specificity can also impact the accuracy and reproducibility of results (Schrohl et al., 2011; Zhang et al., 2021). The VENTANA PATHWAY anti-HER2/neu (4B5) rabbit monoclonal primary antibody has been approved by the FDA as the first companion diagnostic test to identify HER2-low mBC patients for trastuzumab deruxtecan (T-DXd) targeted therapy (Zhang and Peng, 2022). However, assessing the reliability of other commonly used HER2 IHC testing methods is still pending (Zhang and Peng, 2022). Repeating the test for equivocal results may help to exclude technical problems, however, it does not always lead to a definitive result. It should be noted that the HER2 IHC testing was initially designed to distinguish high levels of HER2 expression (i.e., almost 2 million molecules/cell which corresponds to HER2 IHC 3+) from lower levels of HER2 expression (i.e., 20,000 to 500,000 molecules/cell for HER2 IHC 0 to 2+). Therefore, this method has not been developed for detecting HER2-low tumors (Zhang and Peng, 2022). On the other hand, in the post-analytical phase, inter-observer variability can occur due to the lack of consistent epithelial internal positive control for HER2 in non-neoplastic breast tissue (Sajjadi et al., 2022; Zhang et al., 2022). The subjective mode of HER2 assessment and HER2 intratumoral heterogeneity in HER2-low breast cancer are other barriers with great influence on HER2-low assessment (Bianchi et al., 2015; Jensen et al., 2020; Zhang et al., 2020). To determine the suitability of the current standard HER2 IHC assays for identifying patients with HER2-low breast cancers, a concordance survey of 18 pathologists reading 170 breast cancer biopsies was carried out (Fernandez et al., 2022). Not surprisingly, the concordance for IHC scores between score 0 and score 1+ was lower compared to score 2 + vs. score 3+ (26% and 58% concordance, respectively). This difference is likely related to the fact that before the T-DXd approval the distinction between score 0 and score 1+, unlike score 2+ and score 3+, was not related to true clinical implications (Fernandez et al., 2022). It should be noted that almost 50% of patients with breast cancer have HER2-low disease (Banerji et al., 2019; Tarantino et al., 2020). This inaccuracy in the real-world clinical practice could lead to misassignment of a great number of potential candidates for ADC therapies (Yue et al., 2021).

**TABLE 2:** Summary table listing the current challenges and future directions to improve HER2-low scoring.

Phase	Current challenges	Possible solutions
Pre-analytical	Variables influencing the IHC staining intensity: fixation, antigen retrieval, reaction time, temperature, and substrate concentration	Implementation and follow-up of strict SOPs, describing precisely the workflow
		Repeating the test for equivocal results may help to exclude technical problems
	The availability of different antibody clones with varying specificity	Updated guidelines that assess the reliability of commonly used HER2 IHC testing methods
Analytical	The subjective mode of HER2 assessment, observer variability	Rigorous quality control procedures and well-defined guidelines based on amplified clinical trials and patient recruitment to further educate pathologists for higher concordance in scoring
		Implementation of methodologies to improve HER2 assessment including machine learning approaches
	The heterogeneity of HER2 expression and/or amplification	Recruitment of further diagnostic approaches with a more precise cut-off
		Further investigation on whether the presence of intratumor heterogeneity may affect the efficacy of ADCs

Abbreviations: IHC, immunohistochemistry; SOPs, standard operating procedures; HER2, human epidermal growth factor receptor 2; ADC, antibody drug conjugate.

The heterogeneity of HER2 expression and/or amplification is another important challenge that should be considered for improving HER2-low identification. HER2 heterogeneity could be seen in both HER2 gene copy number and/or protein expression (Brunelli et al., 2009; Seol et al., 2012). The American Society of Clinical Oncology (ASCO)/College of American Pathologists (CAP) guidelines have clearly defined intra-tumor heterogeneity of HER2 amplification (i.e., HER2/CEP17 signal ratios >2.2 in 5%–50% of the neoplastic cells). However, there is a lack of consensus to define HER2 heterogeneity for IHC (Ahn et al., 2020; Sajjadi et al., 2022). It should be noted that intratumoral HER2 IHC heterogeneity (i.e., uneven distribution of HER2 expression or different intensities of HER2 staining in tumor cells) is more frequent in the HER2-low (2 + or 1 + IHC score) samples (Seol et al., 2012; Wu et al., 2023a). This condition may contribute to the poor consistency of HER2-low interpretation (Wu et al., 2023a). Moreover, whether the presence of intratumor heterogeneity may affect the efficacy of ADCs is yet to be further investigated (Wu et al., 2023b). The misdiagnosis of HER2-low cases influences directly the prognostic and therapeutic implications of ADCs. Hence, urge the need for rigorous quality control procedures and well-defined guidelines to further educate pathologists for higher concordance in scoring (Sajjadi et al., 2022). Implementation and follow-up of strict standard operating procedures (SOPs), describing the diagnostic workflow from the specimen excision to HER2 report is pivotal (Cree et al., 2014). The invention and application of improved diagnostic methods that are more precise and reproducible may lead to improved responses for a larger number of patients (Ivkovic-Kapic et al., 2019; Angerilli et al., 2021).

## Novel complementary methodologies and future directions

Updates in current guidelines, and advances in methodologies for a more precise measurement could pave the way to ensure consistent and accurate HER2-low identification (Wu et al., 2023b). Clinical trials and patient recruitment are fundamental to improving diagnostic sensitivity for HER2-low breast cancer. In this respect, DESTINY-Breast06 (NCT04494425) is an ongoing phase 3, randomized, multicenter, open-label study on T-DXd vs. chemotherapy in HER2-low, hormone receptor-positive breast cancer patients with disease progression on endocrine therapy in the metastatic setting. In this trial HER2-low tumors (IHC2+/ISH-, IHC 1+, or IHC >0 < 1+) are determined by central laboratory testing results (Wu et al., 2023b). The findings of such trials could contribute to the understanding of clear-cut limits of HER2 expression required for ADC treatment strategy using the available diagnostic techniques (Wu et al., 2023b; Fan and Xu, 2023). Yet, to develop a robust definition of HER2-low, the number of recruited patients in such randomized clinical trials needs to be amplified (Atallah et al., 2022). Recruitment of further diagnostic approaches with a more precise cut-off could facilitate the detection of patients suitable for targeted therapies. For instance, Muotafi et al. developed an assay that is complementary to the conventional methods for the identification of HER2-low unamplified tumors through a combination of quantitative immunofluorescence and mass spectrometry (Moutafi et al., 2022). This method measured absolute amounts of HER2 protein on conventional histology sections where HER2-low expression ranged between 2 and 20 attomol/mm2. By applying this assay to 364 breast cancers, they found that 67% of the tumors had HER2 expression above the limit of quantification and below the level seen in HER2-amplified breast cancer. However, this assay can distinguish between HER2 gene amplified from unamplified. To fully span the dynamic range of HER2 expression more than one assay is needed (Moutafi et al., 2022). Other studies have shown strong agreement between multiple-reaction monitoring mass spectrometry (MRM-MS) results and IHC/ISH, and also demonstrated a correlation between MRM-based measurements and clinical response to HER2-targeted therapy (Steiner et al., 2015; Nuciforo et al., 2016). Kennedy et al. used immunoaffinity-enrichment coupled immuno-MRM-MS to quantify HER2 protein expression in 96 frozen and 119 FFPE breast cancer samples. By examining the agreement of HER2 normalized by glyceraldehyde-3-phosphate dehydrogenase (GAPDH) (as a surrogate for tumor cellularity) to HER2 status, they found an excellent agreement between the GAPDH-normalized immuno-MRM-MS measurements of HER2 and HER2 status (IHC ± ISH) in both the FFPE (*p*-value = 1.09 e−16 by Mann-Whitney test) and frozen (*p*-value = 1.14 e−7 by Mann-Whitney test) tissues. In addition, the immuno-MRM-MS assay showed good sensitivity and specificity in both FFPE (AUC = 0.971) and frozen (AUC = 0.962) samples. Hence, the findings showed this method enables precise, relative quantification of HER2 in HER2-low and HER2-negative tumors (Kennedy et al., 2021). Other methodologies to improve HER2 assessment include machine learning approaches. Increasingly, pathological diagnoses are aided by artificial intelligence (AI), particularly in image processing and quantitative assessments (Koopman et al., 2019; Zakrzewski et al., 2019). Through AI, it is possible to create a computer algorithm that can analyze images, count the amount of HER2 membrane staining, and generate reliable and reproducible results (Koopman et al., 2019; Zakrzewski et al., 2019). Despite great achievements in assessing HER2-positive and -negative tumors, only a few studies have utilized AI for differentiating HER2 0 from 1 + tumors. Palm et al. evaluated an AI-assisted workflow that involved both IHC and ISH for determining the HER2 status of primary and metastatic breast cancer as well as assessing its performance on HER2-low breast tumors. For this, a preliminary cohort of primary breast carcinomas (n = 495), and a study cohort of 67 primary breast carcinomas and 30 metastatic deposits were evaluated for HER2 status by IHC and ISH. After having built the ground truth by practicing breast pathologists, the results of the AI digital image analysis were provided to them and the slides were reassessed. The findings showed that HER2 IHC and its interpretation were troubled by low inter-rater agreement, particularly in the 0 and 1 + range. The IHC/ISH combination was suitable for the detection of amplified and non-amplified HER2 tumors (Cohen’s *κ* = 0.94) however limitations in the classification of HER2 low tumors were observed. While a combined IHC/ISH digitalized AI-assisted workflow for HER2 status determination in primary and metastatic breast cancer including the HER2 low tumors was feasible (Palm et al., 2023). Another recent research has reported of digital image analysis (DIA) efficacy compared to assisted reading (AR) in 761 tumors from 727 patients, IHC stained for HER2 (Sode et al., 2023). The authors reported moderate agreement of 73% (*κ*: 0.55) between DIA and AR, being mostly discordant in cases with heterogeneous and aberrant staining, representing a major setback in the evaluation of HER2. Of note, pathologists evaluated fewer tumors as HER2-1+ and more in the HER2-0 and HER2-2+ categories compared with DIA when looking at the HER2-low category, mostly downgrading the HER2 score to HER2-0 compared to DIA HER2-1+. Thus, authors suggest that pathologist will remain the most significant factor in HER2 evaluation in the near future (Sode et al., 2023). A multi-institutional study showed improved accuracy of HER2 0 and 1 + tumor interpretation when pathologists were assisted by AI compared to not being assisted (0.93 vs. 0.8). Importantly, the accuracy was significantly improved even with the presence of heterogeneity (0.68–0.89) (Wu et al., 2023a). Given the complications of HER2 evaluation in the presence of heterogeneity and the higher prevalence of HER2 heterogeneity in HER2 1 + and 2 + than in HER2 3 + tumors (Seol et al., 2012), this methodology could assist pathologists in a more precise HER2-low evaluation (Wu et al., 2023a). Gustavson et al. used deep learning-based image analysis to generate a novel HER2 Quantitative Continuous Score (QCS). The results showed a high correlation between QCS obtained from automatically detected membranes and those annotated by pathologists (R = 0.993). Moreover, HER2 QCS results had a broad quantitative overlap between IHC and ISH (Gustavson et al., 2021). The use of the artificial neural network (ANN) models that can learn and shape non-linear and complex relationships has been implemented to assist in refining the HER2‐low definition (Atallah et al., 2022). Atallah et al., developed a model by implementing as input the HER2 scoring parameters (i.e., intensity and its distribution, completeness, and percentage of positive cells and cytoplasmic staining) and as an output the HER2 mRNA‐based clusters variable. The ANN model configured HER2 IHC score 1 + as membranous staining in invasive cells either as faint intensity in ≥20% of cells regardless of the circumferential completeness, weak complete staining in ≤10%, weak incomplete staining in >10% and moderate incomplete staining in ≤10%. This model showed statistical significance with Oncotype DX scores and a perfect intra- and inter-observer agreement (kappa value = 0.8 and 0.9, respectively) (Atallah et al., 2022). Such methodologies have shown promising outcomes for a more accurate and reproducible HER2-low assessment compared to the currently available methods. Yet, further investigation to tailor these novel approaches as cost effective and easily accessible assets, as well as performing extensive analytic and clinical validation are necessary prior to introducing them in the real-life clinical practice. Consecutively, the significance of continuous education, updates, quality controls, and multidisciplinary discussions should not be underestimated for optimal patient management.

## Discussion

To date, IHC-ISH combined test is the gold standard approach for assessing HER2 in breast cancer. However, this method suffers from high inter- and intra-observer variability, with the results being conditioned by subjective factors. Moreover, the heterogeneity of HER2 expression and/or amplification adds a layer of complexity to a precise and reproducible HER2-low assessment. To this end, the implementation of clear guidelines, careful supervision of preanalytical and analytical issues, and specialized training for accurate HER2 testing play a crucial role in this process. Moreover, the recruitment of novel diagnostic approaches with a more precise cut-off is essential to define patients who are eligible for targeted therapies specifically ADCs in HER2-low tumors. Among alternative methods, AI and machine learning-based predictors have shown promising results in terms of speed, accuracy, and cost-effectiveness and are likely to play an increasingly important role in this field in the future. It is important to note that AI algorithms are not yet widely used in the clinical setting for the identification of HER2-low breast cancer, and further research is required to validate their accuracy and reliability. However, the potential benefits of AI in this area are significant, and there is ongoing work to develop and refine these algorithms to improve patient outcomes. Introducing such novel approaches in the real-life clinical practice requires extensive analytic and clinical validation. Further investigation including multidisciplinary clinical studies on identifying this fairly large group of patients through novel methodologies with the implementation of strict guidelines, is warranted.
